# Identification and Characterization of Novel Umami and Umami-Enhancing Peptides from Soy Sauce Using an In Silico Approach and Electronic Tongue

**DOI:** 10.3390/foods15040680

**Published:** 2026-02-12

**Authors:** Ting Cai, Zhiqing Lin, Tianliang Wu, Ying Su, Huan Liu, Shiqi Chen, Long Ding

**Affiliations:** 1 College of Food Science and Engineering, Northwest A&F University, Xianyang 712100, China; cting3399@nwafu.edu.cn (T.C.); zhiqinglin@nwafu.edu.cn (Z.L.); wutianliang@nwafu.edu.cn (T.W.); ying.su@nwafu.edu.cn (Y.S.); 2 Chongqing Institute for Food and Drug Control, Chongqing 401121, China; liuh985@163.com; 3 Key Laboratory of Condiment Supervision Technology, State Administration for Market Regulation, Chongqing 401121, China

**Keywords:** umami peptide, umami-enhancing peptide, soy sauce, electronic tongue, molecular docking, molecular dynamics simulations

## Abstract

The objective of the study was to discover novel umami and umami-enhancing peptides in soy sauce and to elucidate the molecular mechanism underlying their umami-enhancing effect. Soy sauce peptides were isolated by resin adsorption and ethanol elution, identified by UPLC-MS/MS, and screened with multiple virtual tools. Results showed that 159 peptides were identified, and four of them, GAAGAAD, HQADGKS, GDDDEVEAAM, and MPPTEPECEK, were predicted to have a potential umami taste. Subsequently, the electronic tongue results suggested GAAGAAD having the strongest umami taste, followed by MPPTEPECEK, GDDDEVEAAM, and HQADGKS. Moreover, GAAGAAD, HQADGKS, and MPPTEPECEK all enhanced umami in sodium glutamate solution, with GAAGAAD showing the most potent effect, increasing umami intensity by 22.3%. Molecular docking revealed that GAAGAAD exhibited a lower binding energy when docked to the T1R1/T1R3–Glu complex compared to T1R1/T1R3 alone, mainly due to the production of more hydrogen bond interactions to enhance its stability, which may be the reason for its umami-enhancing effect. Furthermore, the peptide enhanced the stability of the receptor-Glu complex, potentially explaining its ability to enhance the umami of Glu. This work provides mechanistic insight into the enhancement of umami by soy sauce peptides, highlighting their potential as ingredients for seasoning formulations.

## 1. Introduction

Umami is considered the “fifth basic taste” after sour, sweet, bitter, and salty [1]. It not only enhances richness and smoothness but also reduces sourness and masks a food’s peculiar smell [2]. It has been indicated that umami peptides, organic acids, and nucleotides in food are the key components that produce umami taste [3]. Among these, umami peptides have garnered significant interest for their rich taste profiles and favorable functional properties in food processing [4]. In addition, compared with monosodium glutamate (MSG), umami peptides are considered natural and safe, with better bioavailability, making them promising umami-enhancing agents [5]. Umami peptides, as taste substances derived from natural sources, align more closely with modern consumers’ pursuit of health and safety.

Soy sauce is widely valued for its rich and distinctive umami taste, to which umami peptides contribute significantly [6]. In recent years, several peptides, such as LPEEV, AQALQAQA, and EQQQQ, have been found in soy sauce and demonstrated to possess umami properties [7]. However, due to differences in production methods, microbial fermentation yields a diverse array of peptide types and quantities [8]. Consequently, the continued discovery and study of novel umami peptides are essential for unraveling the complexity of soy sauce’s unique flavor.

Soy sauce umami peptide screening has traditionally relied on sensory evaluation, a method characterized by considerable time and cost requirements, which impedes the rapid discovery of new peptide candidates. Although electronic tongue systems provide an objective alternative for taste profiling, their practical use remains limited in high-throughput contexts due to analogous constraints in duration and operational expenses [9,10]. Recently, virtual screening has been widely used to identify peptides at a lower cost, with greater accuracy and speed [11]. Multiple machine learning tools were employed because they are built on distinct algorithmic principles and training datasets, leading to variations in predictive focus and reliability. Their combined use enables cross-validation, thereby enhancing the reliability and precision of umami peptide screening. Machine learning models have become a prominent approach for predicting umami peptides by analyzing peptide sequences and amino acid composition. For instance, tools such as iUmami-SCM and UMPred-FRL are designed to identify potential umami peptides based on sequence features. Similarly, models such as Umami-MRNN and Umami_YYDS leverage advanced algorithms to enhance prediction accuracy from both structural and functional perspectives [4,10,12]. The four prediction models were combined to screen for potential umami peptides in *Flammulina filiformis* [13]. However, in studies of soy sauce peptides, only sequence-based machine learning predictors, such as iBitter-SCM, iBitter-Fuse, and BERT4Bitter, were used to predict peptide bitterness [14]. The study for screening soy sauce umami peptides using virtual tools has not been well reported.

Some umami peptides have been found to exhibit umami-enhancing effects when interacting with other umami substances, thereby increasing umami perception intensity compared to when used individually [15]. For instance, the peptides ALPEEV, EAGIQ, and LPEEV from soy sauce showed enhanced umami activity [7]. The peptides DGV, AD, DR, DK, SGDAW, and NDDGW have been shown to have umami flavor and to potentiate the umami of MSG solution [16]. Similarly, the peptide AEEHVEAVN demonstrated to potentiate the umami intensity of chicken soup [17]. In addition, emerging evidence indicates that specific umami peptides may modulate salt taste perception, thereby enhancing overall saltiness perception in food matrices [15]. Therefore, umami-enhancing peptides can reduce sodium content while maintaining umami taste and are essential for the development of low-sodium foods in food processing. To further explore its mechanisms, molecular docking and molecular dynamics simulation (MDS) were performed to analyze the interactions among the umami peptide, MSG, and the taste receptors T1R1/T1R3. This method has been applied to investigate the umami-enhancing peptides of *Flammulina filiformis* and *Russula vinosa Lindblad* [13,15]. Moreover, research suggests that umami peptides intensify the umami of MSG solutions by binding to the open conformation of the T1R3 receptor, which is adopted upon its activation by MSG [18,19]. Analysis of peptides extracted from Sanhuang chicken revealed that these components could enter the Venus flytrap (VFT) domain of the T1R3 receptor. Residues Arg303, Ser123, and His121 were further identified as key binding sites that may play an essential role in regulating the umami-enhancing effect [20]. So far, few studies have examined the mechanisms by which umami peptides enhance umami [13,15], and the mechanisms of soy sauce umami peptides have not been well reported, leaving the underlying mechanisms unclear.

In our previous study, soy sauce peptides were isolated by resin adsorption followed by ethanol elution at 0%, 20%, 40%, and 60% (*v*/*v*). The peptide fraction eluted with 60% ethanol had the highest umami intensity [6]. Accordingly, the objective of this work was to discover and characterize novel umami peptides and umami-enhancing peptides from the 60% ethanol-eluted soy sauce peptide fraction, and to explore the molecular mechanism of umami taste. In this study, peptide sequences of the 60% ethanol-eluted soy sauce peptide fraction were identified using ultra-high-performance liquid chromatography–mass spectrometry (UPLC-MS/MS). The candidate peptides with umami were predicted and screened by an in silico approach with multiple virtual tools. Then, the umami and umami-enhancing tastes of these peptides were assessed utilizing an electronic tongue system, followed by molecular docking and MDS to investigate the umami mechanism.

## 2. Materials and Methods

### 2.1. Materials and Chemicals

Soy sauce was acquired from a local supermarket located in Yangling, Shaanxi province, China. The XAD-16 macroporous resin was obtained from Aladdin Biochemical Technology Co., Ltd. (Shanghai, China). The peptides with a purity of over 96% were synthesized via the Fmoc solid-phase method by China Peptides Biochemical Co., Ltd. (Hangzhou, Zhejiang, China).

### 2.2. Preparation of Peptides of Soy Sauce

The soy sauce peptides (SSPs) were isolated by resin adsorption and ethanol elution [6]. Briefly, the resins were initially activated using 95% (*v*/*v*) ethanol and subsequently dried at a temperature of 60 °C. Then, the dried resin (5 g) was immersed in 95% (*v*/*v*) ethanol for activation for 12 h, after which 95% ethanol was removed by washing with distilled water. Subsequently, the soy sauce (100 mL) was added to the resin and adsorbed in an incubator (30 °C, 10 h, 150 r/min) and then sequentially eluted by 300 mL of 0%, 20%, 40%, and 60% (*v*/*v*) ethanol, with each elution lasting 12 h under constant agitation at 150 r/min. Following the collection, the eluted fractions were lyophilized. The obtained SSP samples were further stored at −20 °C.

### 2.3. Identification of SSPs by UPLC-MS/MS

The sample was first desalted using a Pierce C18 centrifugal column (10 µL; Thermo Fisher Scientific, Waltham, MA, USA), then analyzed by UPLC-MS/MS (Orbitrap Fusion Lumos, Thermo Fisher Scientific) [21]. The SSPs were dissolved in the mobile phase A (0.1% formic acid in water), and analyzed using a nanoflow UPLC system with a pre-column and an analytical nano-column (Acclaim PepMap RPLC C18, Thermo Fisher Scientific, 150 μm × 150 mm, 1.9 μm, 100 Å). The SSPs were eluted with a linear gradient of mobile phase B (0.1% formic acid and 80% acetonitrile) at a flow rate of 600 nL/min. The gradient elution was set as follows: mobile phase B from 6 to 9% for 5 min, 9–14% for 15 min, 14–30% for 30 min, 30–40% for 8 min, and 40–95% for 2 min. In addition, the mass spectrometry (MS) conditions were as follows: scan range of 375–1500 m/z, AGC target of 4.0 e5, Orbitrap resolution of 60,000, and HCD collision energy of 30%. Finally, the raw MS file was analyzed and searched using the Thermo Proteome Discoverer software (Thermo Fisher Scientific, Waltham, MA, USA), combined with the UniProt soybean database.

### 2.4. Screening of Potential Soy Sauce Umami Peptides (SSUPs)

The potential SSUPs were screened by an in silico approach, based on previously established methods [22,23]. Several computational models, including iUmami-SCM, UMPred-FRL, Umami_YYDS, and Umami-MRNN, were employed to predict and screen umami peptides. Moreover, potential peptide toxicity was evaluated using ToxinPred (https://webs.iiitd.edu.in/raghava/toxinpred/, accessed on 1 March 2025), while allergenicity was assessed with AllerTOP (https://www.ddg-pharmfac.net/AllerTOP/, accessed on 1 March 2025).

### 2.5. Electronic Tongue Evaluation

#### 2.5.1. Evaluation of the Umami Properties of Peptides

The umami characteristics of the synthesized peptides were evaluated using the SA402B electronic tongue system (Intelligent Sensor Technology, Inc., Atsugi, Japan) as previously described [17]. The SA402B electronic tongue can determine the sourness, bitterness, astringency, umami, saltiness, aftertaste-B, aftertaste-A, and richness of peptides. Briefly, the 7 mg synthesized peptides were dissolved in distilled water (70 mL) to prepare 0.10 mg/mL solutions. Before testing, the sensor and the reference electrode were activated for 24 h. The measurement cycle comprised a 30 s taste collection phase, followed by 30 s for aftertaste collection and 30 s for signal stabilization. Subsequently, a 336 s cleaning step was performed. Each sample underwent four consecutive test cycles, with data from the final three cycles retained for analysis.

#### 2.5.2. Evaluation of the Umami-Enhancing Effect of Peptides

The 0.2 mg/mL synthetic peptide samples were mixed with 0.2 mg/mL MSG solution at a 1:1 ratio, and their umami-enhancing effects were evaluated using the SA402B electronic tongue. The MSG solution of 0.1 mg/mL was used as the reference. The synthetic peptide samples were tested four times, and the data from the last three tests were used for analysis.

### 2.6. Homology Modeling of T1R1/T1R3

The umami receptor T1R1/T1R3 was constructed by homology modeling, following a previous method [24]. The sequences of the human taste receptors T1R1 (UniProt ID: Q7RTX1) and T1R3 (UniProt ID: Q7RTX0) were all retrieved from the UniProt database (https://www.uniprot.org/, accessed on 9 March 2025). The crystal structure of medaka fish sweet receptor T1R2a-T1R3 (PDB: 5 × 2 M) was selected as a structural template for constructing the T1R1/T1R3. The SwissModel website (https://swissmodel.expasy.org/interactive, accessed on 15 March 2025) was employed for generating the homology model based on template 5X2M and target sequences (T1R1 and T1R3). Finally, the model reliability was evaluated using the Ramachandran plot analysis obtained via the SAVESv 6.0 online platform (https://saves.mbi.ucla.edu/, accessed on 18 March 2025).

### 2.7. Molecular Docking

#### 2.7.1. Molecular Docking of Umami Peptides with T1R1/T1R3

To investigate the umami mechanism of these peptides, molecular docking was performed using a previously established method with minor adjustments [25]. The molecular structures of these peptides were generated and initially optimized through ChemDraw and Chem3D (version 20.0), followed by further energy minimization using the MMFF94s force field in Avogadro (version 1.99) to obtain a stable structural conformation. The AutoDock tools software (version 1.5.6) was used to optimize the T1R1/T1R3 structure by adding nonpolar hydrogen and removing crystalline water. Similarly, the peptides’ structures were optimized by adding nonpolar hydrogen atoms. Then, the peptides were docked into the T1R1/T1R3 receptor employing AutoDock Vina, and the most optimal binding conformation was selected based on the energy scores. Moreover, the docking pocket grid size and the active center coordinate value were set as follows: for T1R1: center of X = 40.845, Y = 30.808, Z = 43.536, size X = 40, Y = 40, Z = 40; for T1R3: center of X = 49.528, Y = 37.528, Z = 7.194, size X = 40, Y = 40, Z = 40. Finally, visualization and analysis of the docking results were performed with PyMol (Schrödinger, Inc., New York, NY, USA).

#### 2.7.2. Molecular Docking of Umami Peptides with T1R1/T1R3-Glu Complex

To further investigate the mechanism by which peptides enhance the umami taste of MSG, molecular docking was performed based on a previous research method with appropriate modifications [26]. Briefly, Glu was first docked to the T1R1 and T1R3 receptors, and the conformation exhibiting the minimal binding energy was chosen to construct the T1R1-Glu and T1R3-Glu receptor complexes, respectively. Then, the T1R1-Glu and T1R3-Glu receptor complex was subjected to further docking analysis with the peptides using AutoDock Vina.

### 2.8. MDS

The receptor–ligand complexes derived from the docking results were further analyzed by the MDS method, as previously described [27]. Briefly, the proteins and ligands in the docked complexes were separated using Pymol software (Schrödinger, Inc., New York, NY, USA) and exported as individual PDB-format files. The T1R1/T1R3 receptor topology was generated through the Amber99SB force field, while the ligand topologies were created through the GAFF force field. Next, water solvent was added to the receptor–ligand system using the TIP3P water model. To maintain electrostatic neutrality, sodium and chloride ions were added to the system. The receptor–ligand system was subjected to energy minimization for 50,000 steps. Then, the protein–ligand system was brought to equilibrium under isothermal NVT conditions and isobaric NPT conditions over 100 ps. Ultimately, the 100 ns of MD were performed at ambient conditions.

### 2.9. Statistical Analysis

The data of umami and umami-enhancing taste were expressed as mean ± SD (n = 3). Statistical analysis was executed using GraphPad Prism 9.5 (GraphPad Corporation, San Diego, CA, USA). Differences between groups were assessed using one-way analysis of variance (ANOVA) and Duncan’s post hoc test, with *p* < 0.05 considered statistically significant.

## 3. Results and Discussion

### 3.1. Identification and Virtual Screening of Umami Peptides from Soy Sauce

**Figure 1:**
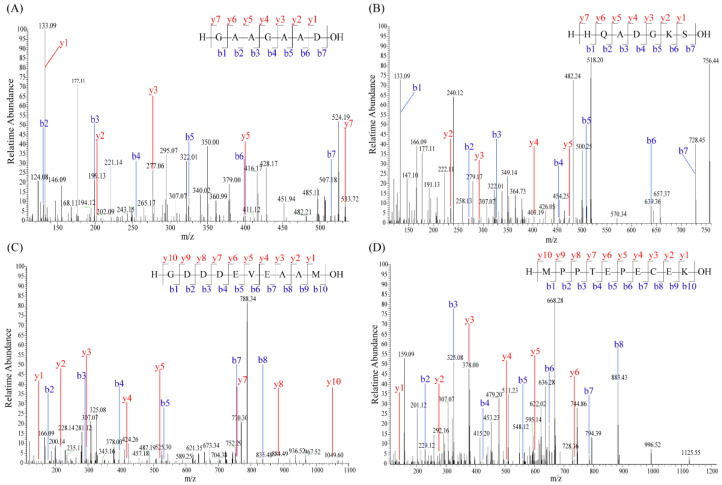
The MS/MS spectrum of the soy sauce peptides. (**A**) GAAGAAD, (**B**) HQADGKS, (**C**) GDDDEVEAAM, and (**D**) MPPTEPECEK.

Based on our earlier research, the four SSP fractions, including SSP-0%, SSP-20%, SSP-40%, and SSP-60%, were obtained by XAD-16 macroporous resin adsorption and elution with 0%, 20%, 40%, and 60% (*v*/*v*) ethanol, respectively. The SSP-60% fraction exhibited the highest umami intensity, as measured by the electronic tongue [6]. However, the specific umami peptide sequences remained unknown. Therefore, the peptides of SSP-60% fraction were further identified and characterized. A total of 159 soy sauce peptides were successfully identified through UPLC-MS/MS. It has been shown that the peptides whose length is not more than ten amino acid residues might have umami taste [28,29]. Among the 159 peptides, there were 22 peptides with a length of ten amino acids or fewer to be selected for further investigation (Table S1). Then, a machine learning-based online tool for predicting umami was used to rapidly screen potential soy sauce umami peptides (Figure S1). In general, the peptides with iUmami-SCM scores > 588, UMPred-FRL probability > 0.5, Umami_YYDS probability > 0.9, and characteristic of umami in Umami-MRNN have been well demonstrated as umami peptides [30,31,32,33]. In particular, Umami-MRNN has been reported to achieve approximately 93% accuracy in predicting umami taste, while iUmami-SCM and UMPred-FRL have demonstrated accuracies of around 88.9% and 86.7%, respectively [34,35,36]. Furthermore, the toxicity and allergenicity of peptides are also essential for screening umami peptides to ensure human health and safety [37,38]. In this research, the four potential umami peptides, GAAGAAD, HQADGKS, GDDDEVEAAM, and MPPTEPECEK, were finally selected, and their identification results are presented in Figure 1. Moreover, it is known that Asp (D) and Glu (E) serve a key role in umami taste. And a lot of umami peptides contain one or more D and E residues [28]. Interestingly, the predicted four peptides all contained D or E residues with frequencies ranging from 14.29% to 50% (Table S1).

### 3.2. Umami Taste Characteristics of Synthesized Peptides

**Figure 2:**
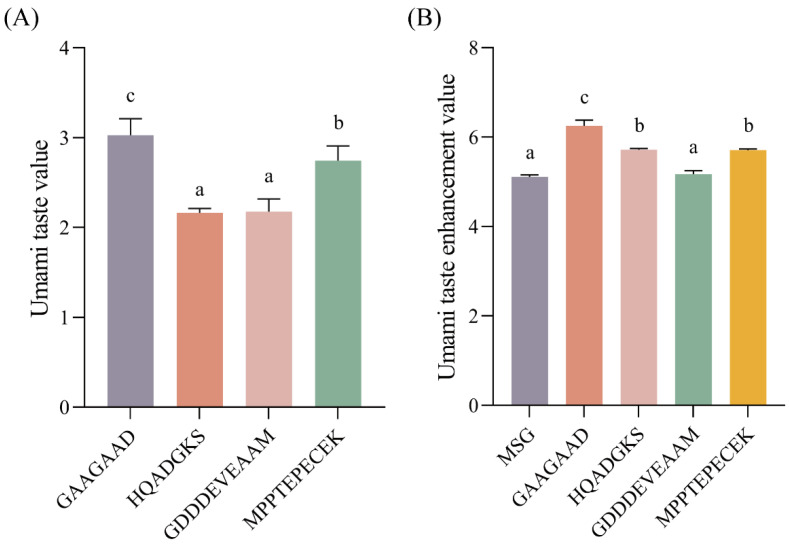
Umami (**A**) and umami-enhancing taste (**B**) of the peptides. Different letters indicate statistically significant differences (*p* < 0.05).

To validate whether the peptides selected by an in silico approach possess umami intensity, the four peptides GAAGAAD, HQADGKS, GDDDEVEAAM, and MPPTEPECEK were synthesized, and the umami taste characteristics were evaluated using the SA402B electronic tongue. It was found that all four peptides showed umami taste (Figure 2A), consistent with the results of the virtual screening. Among them, the peptide GAAGAAD (3.03) exhibited the highest value of umami, followed by MPPTEPECEK (2.74), GDDDEVEAAM (2.17), and HQADGKS (2.16) (*p* < 0.05). In addition, these peptides also showed bitterness, sourness, astringency, and saltiness taste (Figure S2A). The acidic amino acids might play a certain role in the sourness and astringency of these peptides. In contrast, the bitterness might be attributed to the hydrophobic amino acids, including glycine (Gly, G), phenylalanine (Phe, F), and proline (Pro, P) [29,39]. Therefore, this study is the first to discover four new types of umami peptides in soy sauce, namely peptides GAAGAAD, HQADGKS, GDDDEVEAAM, and MPPTEPECEK, which have not been reported previously [40,41]. These findings deepen the molecular understanding of soy sauce flavor and provide new candidates for natural umami agents in food development.

To further investigate whether these peptides enhance the umami of MSG solution, the umami characteristics of the mixed solutions of peptide solution and MSG solution were evaluated by the SA402B electronic tongue system. The results of Figure 2B suggested that the peptides GAAGAAD, HQADGKS, and MPPTEPECEK significantly enhanced the umami of MSG solutions (*p* < 0.05), while GDDDEVEAAM showed no significant effect (*p* > 0.05). This result indicated that the peptides GAAGAAD, HQADGKS, and MPPTEPECEK all revealed umami-enhancing effects with MSG. The peptide GAAGAAD had the highest umami-enhancing effect, with an enhancement rate of 22.31%, followed by MPPTEPECEK and HQADGKS, both with 11.74%. Similarly, the peptides LKDGQAQ, QISPYRRI, ISWCFTY, LLMLVV, and VPAFLRQARCFS have been reported to enhance umami intensity in MSG solutions, with umami-enhancing rates exceeding 10% [27]. In addition, in our study, the four peptides significantly increased the taste of salt but reduced the bitter taste of the MSG solution (*p* < 0.05) (Figure S2B). These findings implied that soy sauce umami peptides GAAGAAD, HQADGKS, and MPPTEPECEK could enhance the umami taste of food and reduce salt intake, thereby promoting human health [2]. In addition, the electronic tongue outputs relative intensity values based on the sensor array’s response signal. This value is measured by using a mixed KCl and tartaric acid solution as the reference. It can objectively, stably, efficiently, and safely analyze the overall taste profile of the sample, delivering relative-intensity values for the taste. However, to more directly reflect the overall perception and acceptance of taste in humans, sensory evaluation experiments are still needed to further verify the umami taste of peptides.

### 3.3. Homology Modeling of T1R1/T1R3 Receptors

**Figure 3:**
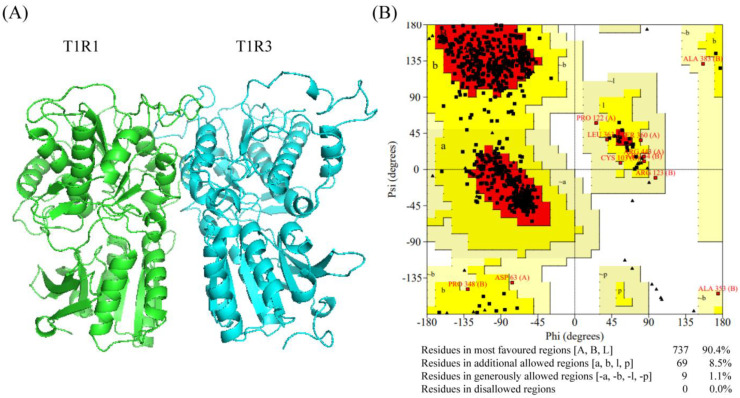
Structure (**A**) and Ramachandran plot (**B**) of T1R1/T1R3.

Before exploring the interaction and mechanism of receptors and peptides, we must determine the structure of the umami receptor. T1R1/T1R3, mGluR1, and mGluR4 are recognized as receptors involved in umami taste perception [42,43]. Among these receptors, the T1R1/T1R3 is considered the primary umami receptor, as it exhibits the strongest binding affinity for umami peptides [44]. It has a large Venus flytrap (VFT) domain that can recognize umami peptides [9,45]. Although the crystal structure of T1R1/T1R3 is unknown, it can be derived via homology modeling [22]. For example, T1R1/T1R3 has been constructed with either the metabotropic glutamate receptor (1EWK) or the 5X2M serving as the structural template [46]. During homology modeling, the template sequence must have more than 30% similarity to the target sequence [29]. In our study, sequence comparison results showed that T1R1 and T1R3 exhibited 34.75% and 37.29% sequence similarity with 5X2M, respectively, using the NCBI BLAST server. However, the T1R1 and T1R3 exhibited 34.34% and 33.55% sequence similarity with 1EWK [47]. Therefore, the T1R1/T1R3 receptor model was constructed using the target sequence (T1R1 and T1R3) and template sequence (5X2M) (Figure 3A).

Moreover, the T1R1/T1R3 model was evaluated via the SAVES v6.0 validation tool [48]. According to Ramachandran plot analysis, the T1R1/T1R3 model was considered reliable, with less than 5% of its amino acid residues located in disallowed regions. In general, the amino acid residues falling within the red region are reliable, those located in the yellow region are considered allowable, and those located in the light yellow region are also within acceptable limits [49]. In our study, Ramachandran plot analysis of the T1R1/T1R3 model showed a favorable residue distribution. Specifically, 90.4% of residues were located in the most favored regions, 8.5% in the additional allowed regions, and 1.1% in the generously allowed regions, all of which contributed to the superior conformational space (Figure 3B) [3,50]. Therefore, the T1R1/T1R3 homology model was considered satisfactory and was subsequently used as the umami receptor to dock peptides.

### 3.4. Analysis of Interactions Between Peptides and T1R1/T1R3

Molecular docking is a computational simulation technique based on bioinformatics for simulating and analyzing interactions between proteins and ligands [51]. Thus, the peptides GAAGAAD, HQADGKS, GDDDEVEAAM, and MPPTEPECEK were docked with the T1R1/T1R3 receptor to investigate their interactions and binding affinities. In general, binding energy is regarded as an indicator of protein–ligand complex stability, with lower values corresponding to higher binding affinity [4]. It is widely observed that peptides exhibiting binding energies within the range of −15 to −5 kcal/mol are more likely to produce umami taste [52]. The binding energies of the GAAGAAD, HQADGKS, GDDDEVEAAM, and MPPTEPECEK ranged from −7.60 to −9.10 kcal/mol in this study (Table S2), confirming the screening and electronic tongue results. Interestingly, the peptides exhibited lower binding energies with T1R3 than with T1R1, indicating that T1R3 was more capable of forming stable complexes with peptides. These results may be due to the T1R3 receptor being in an open state, which facilitates the binding of umami peptides [29]. However, the binding energies of the peptides were not consistent with the umami intensity, suggesting that the binding energies were not directly relevant to the umami of the peptides. Similar results had also been reported elsewhere [53].

**Figure 4:**
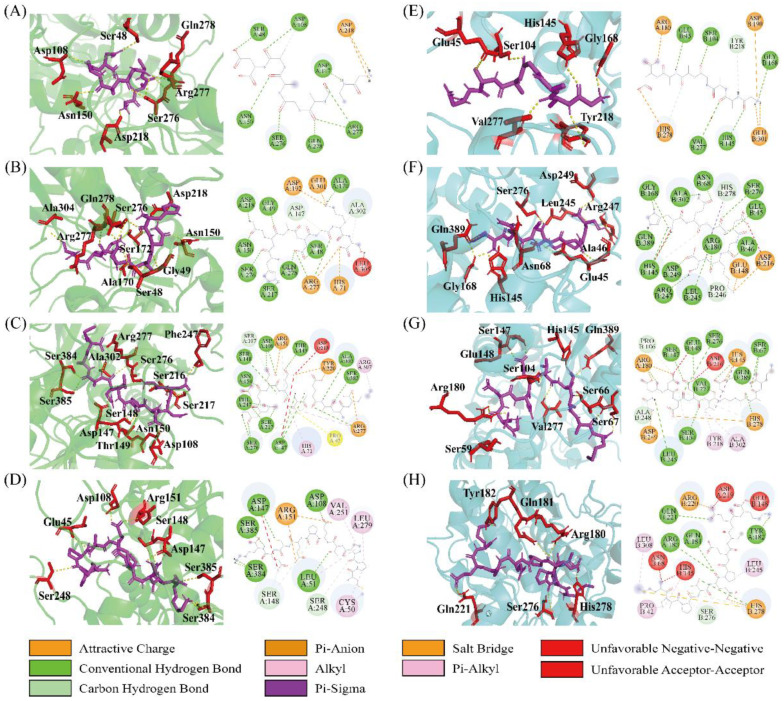
The interactions between T1R1/T1R3 and peptides (left: 3D plot; right: 2D plot). Molecular docking of T1R1 with GAAGAAD (**A**), HQADGKS (**B**), GDDDEVEAAM (**C**), and MPPTEPECEK (**D**). Molecular docking of T1R3 with GAAGAAD (**E**), HQADGKS (**F**), GDDDEVEAAM (**G**), and MPPTEPECEK (**H**).

**Figure 5:**
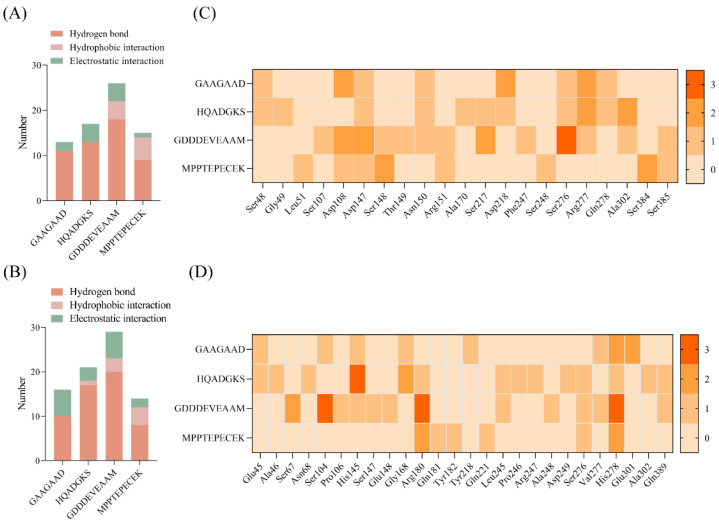
Number of interactions between peptides and T1R1 (**A**) and T1R3 (**B**), and the heatmap of the number of hydrogen bonds between peptides and the key active-site residues of T1R1 (**C**) and T1R3 (**D**).

Furthermore, the binding sites on T1R1/T1R3 and their interactions with the four peptides were analyzed. The predominant interactions between peptides and receptors primarily include hydrogen bonds (Conventional hydrogen bonds, Salt Bridges, and Carbon-hydrogen bonds), hydrophobic interactions (Pi-alkyl, Alkyl, and Pi-sigma), and electrostatic interactions (Attractive charge and Pi-anion). As shown in Figure 4, each of the peptides was able to access the VFT domain within the binding cavity of T1R1/T1R3. It was also observed that hydrogen bonding represented the predominant interaction type, which accounted for a large proportion (55–85%). However, the interactions of hydrophobic and electrostatic forces were relatively low or non-existent (Figure 5A,B). Specifically, peptides GAAGAAD, HQADGKS, GDDDEVEAAM, and MPPTEPECEK were predicted to form 11, 13, 18, and 9 hydrogen bonds with T1R1, respectively (Figure 5A). In contrast, these peptides formed 10, 17, 20, and 8 hydrogen bonds with T1R3, respectively (Figure 5B). It was interesting to find that the number of hydrogen bonds showed a strong negative correlation with binding energy, with correlation values of −0.80 and −0.73 for T1R1 and T1R3, respectively. It was speculated that the hydrogen bond might be a pivotal interaction force in the formation of a stable T1R1/T1R3–peptide complex.

Subsequently, the sites on the receptors that generate the hydrogen bond interaction were further analyzed. The number of 21 amino acid residues on T1R1 was found by docking peptides, of which hydrophilic amino acid sites accounted for 60–100% (Figure 5C). Similarly, 26 amino acid residues on T1R3 were found by docking peptides, of which hydrophilic amino acid sites accounted for 76–100% (Figure 5C). Hence, the hydrophilic amino acid residues on the receptor played a crucial part in recognizing umami peptides [49,54]. Nevertheless, differences in amino acid residues resulting from docking may be due to structural differences and allosteric effects of the peptides [46]. It further found that Ser was the highest frequently occurring amino acid residue on T1R1/T1R3 when docking with umami peptides. For example, Ser48, Ser107, Ser148, Ser217, Ser248, Ser276, Ser384, and Ser385 on T1R1 and Ser67, Ser104, Ser147, Ser276 on T1R3 were all formed by hydrogen bonding with umami peptides (Figure 5C,D). This suggested that the Ser residue in T1R1/T1R3 was critical for peptide binding, consistent with previous findings [17]. In addition, the key residues included Asp108, Asp147, Asn150, Ser276, and Arg277 of T1R1 and His145, Arg180, and Ser276 of T1R3. These residues exhibited high binding frequencies, with at least three peptides docking with the receptor, generating interaction forces at these sites. In addition, the residues including Asp108, Asp147, Asn150, Ser276, and Arg277 of T1R1 and His145 and Ser276 of T1R3 were the key binding residues that have been reported [17,52,55]. However, the molecular docking results presented in this study were computational predictions and therefore required further experimental validation.

### 3.5. The Umami-Enhancing Mechanisms of Peptides

In recent years, the molecular mechanisms by which peptides and MSG interact with umami receptors, as well as the umami-enhancement, have been continuously explored [26]. Previous studies have shown that MSG expands the binding pocket of T1R1/T1R3, thereby enhancing umami taste by facilitating peptide and key residue binding [19]. However, because MSG ionizes in aqueous solution, Glu is often used as an alternative [20]. Therefore, Glu was first docked to T1R1 and T1R3 to generate the T1R1-Glu and T1R3-Glu complex, which served as a model for investigating the molecular mechanisms underlying the peptide’s umami-enhancing effects. These results suggested that the binding energies of Glu docking to T1R1 and T1R3 were all −5.3 kcal/mol, consistent with previously reported values for MSG binding to T1R1/T1R3 [15]. Therefore, it can serve as a reference for evaluating the influence of umami-enhancing peptides on Glu binding. As illustrated in Figure S3A,B, Glu binds to the VFT domain of T1R1 and T1R3. The 3D interaction showed that Glu could bind to the VFT domain of T1R1 and T1R3. Additionally, the interactions between T1R1 and Glu were mediated by hydrogen bonding and electrostatic interactions, while those between T1R3 and Glu involved hydrogen bonds, hydrophobic interactions, and electrostatic forces (Figure S3C). Similarly, hydrogen bonds were the predominant interaction, accounting for the highest proportion. All of those were consistent with the results for umami peptides.

Subsequently, the T1R1-Glu and T1R3-Glu complex was designated as the new receptor to dock with the four peptides. The docking binding energies of GAAGAAD, HQADGKS, GDDDEVEAAM, and MPPTEPECEK ranged from −8.0 to −9.2 kcal/mol (Table S3). On the one hand, the four peptides exhibited lower binding energies with T1R1-Glu and T1R3-Glu compared to T1R1 and T1R3 alone, indicating enhanced stability of the resulting T1R1-Glu-peptide and T1R3-Glu-peptide complexes. This result might explain the peptides’ umami-enhancing effect by increasing Glu’s binding to T1R1/T1R3. This result is consistent with previous studies [13]. On the other hand, the binding energies of peptides with T1R3-Glu were lower than those with T1R1-Glu, aligning with the trend observed for T1R1-peptide and T1R3-peptide interactions.

**Figure 6:**
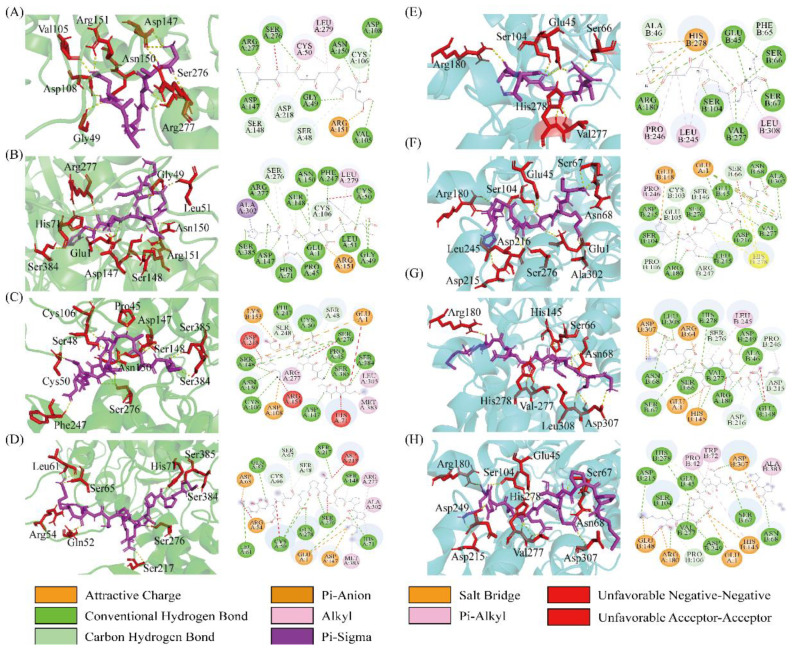
The interactions between T1R1/T1R3-Glu and peptides (left: 3D plot; right: 2D plot). Molecular docking of T1R1-Glu with GAAGAAD (**A**), HQADGKS (**B**), GDDDEVEAAM (**C**), and MPPTEPECEK (**D**). Molecular docking of T1R3-Glu with GAAGAAD (**E**), HQADGKS (**F**), GDDDEVEAAM (**G**), and MPPTEPECEK (**H**).

**Figure 7:**
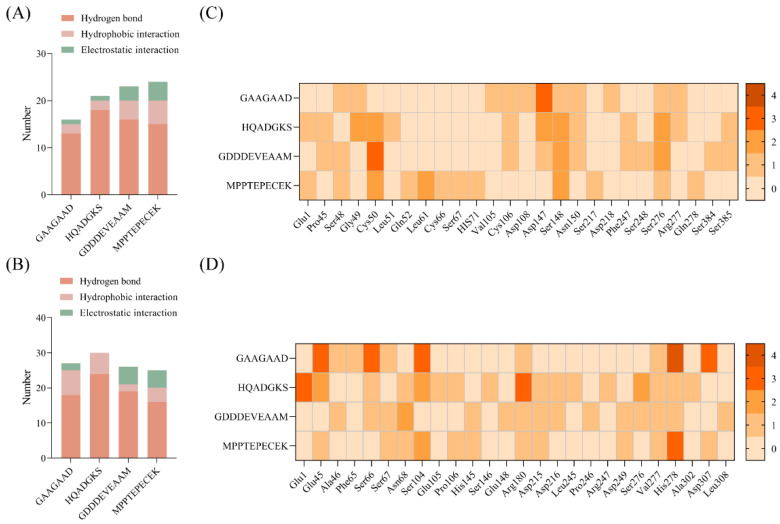
Number of interactions between peptides and T1R1-Glu (**A**) and T1R3-Glu (**B**), and the heatmap of the number of hydrogen bonds between peptides and the key active-site residues of T1R1-Glu (**C**) and T1R3-Glu (**D**).

Furthermore, the interaction analysis of the T1R1/T1R3-Glu receptor with four peptides is presented in Figure 6. Similar to that of T1R1/T1R3, the peptide was also found to enter the VFT domain and form hydrogen bonding, hydrophobic interactions, and electronic interactions with T1R1/T1R3-Glu. Compared with the T1R1/T1R3-Glu complex, the addition of the peptide significantly increased the quantity and diversity of interaction forces. Among them, hydrogen bonds were the main interaction force, with the highest proportion. Interestingly, the quantity of interactions and hydrogen bonds of peptides GAAGAAD, HQADGKS, and MPPTEPECEK with T1R1/T1R3-Glu was all higher than that of T1R1/T1R3, while GDDDEVEAAM exhibited a lower number than that of T1R1/T1R3 (Figure 7A,B). These results might explain that peptides GAAGAAD, HQADGKS, and MPPTEPECEK, but not GDDDEVEAAM, displayed umami-enhancing effects. Similarly, it was also found that Ser was a frequently occurring amino acid residue of T1R1/T1R3-Glu docking to the peptides, including Ser48, Ser67, Ser148, Ser217, Ser248, Ser276, Ser384, and Ser385 of T1R1 and Ser66, Ser67, Ser104, Ser146, and Ser276 of T1R3 (Figure 7C,D). Residues Ser48, Cys50, Cys106, Asp147, Ser148, Asn150, and Ser276 of T1R1-Glu and residues Glu45, Ser66, Ser67, Asn68, Ser104, Arg180, Asp215, Val277, and His278 on T1R3-Glu occurred most frequently in the docking with peptides (Figure 7C,D). Furthermore, in addition to the previously identified key active sites, residues such as Cys50, Cys106, and Ser148 of T1R1 and Ser66, Ser67, Arg180, Asp215, Val277, and His278 of T1R3 might serve as important potential active sites. However, few studies have investigated the umami-enhancing mechanism of peptides using molecular docking of ternary complexes involving the receptor, Glu, and the peptides. Therefore, this study will provide new insights into the molecular mechanism of umami enhancement of peptides.

### 3.6. Molecular Dynamics Simulation

Different from molecular docking, MDS predicts the dynamic behavior of protein–ligand complexes [27,56]. It illustrated the interaction mechanisms between peptides and receptors in an aqueous environment by visualizing their time-dependent behavior [43]. In our present study, peptides GAAGAAD, HQADGKS, and MPPTEPECEK had umami taste and an umami-enhancing effect. To further study the mechanisms of GAAGAAD, HQADGKS, and MPPTEPECEK on umami taste and the umami-enhancing effect, MD simulations of 100 ns were performed for the T1R1/T1R3–peptide and T1R1/T1R3-Glu-peptide complex.

**Figure 8:**
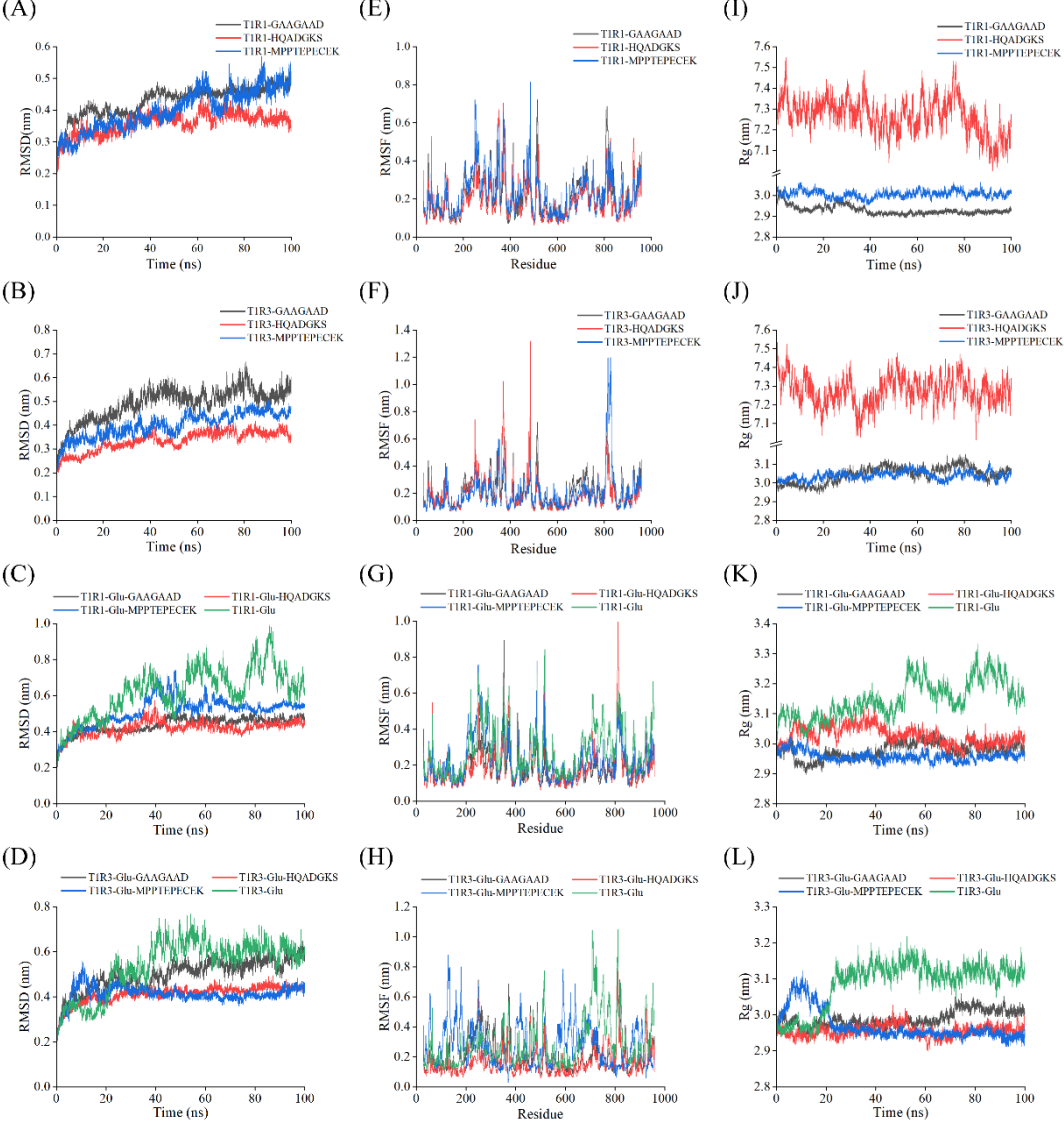
Results of molecular dynamics simulations of the receptor–ligand complex. (**A**–**D**) RMSD; (**E**–**H**) RMSF; (**I**–**L**) Rg.

In general, the RMSD is commonly used to assess the fluctuation range during receptor–ligand docking, with a smoother RMSD curve indicating greater complex stability [33]. In this study, it was found that the RMSD fluctuated strikingly in the first 40 ns but then reached equilibrium and remained stable for the T1R1-peptide complexes and T1R3-peptide complexes (Figure 8A,B). The RMSD of the T1R1-Glu-peptide complexes and T1R3-Glu-peptide complexes fluctuated strikingly in the first 20 ns but then reached equilibrium and remained stable (Figure 8C,D). Compared with the receptor-peptide complex, the receptor-Glu-peptide ternary complex reached equilibrium more quickly, shortening the cycle and demonstrating greater stability. These results indicated that greater complex stability corresponds to stronger umami ability. Furthermore, the RMSD of the T1R1-Glu and T1R3-Glu complexes stabilized after 40 ns, exhibiting fluctuation ranges of 1 nm and 0.8 nm, respectively (Figure 8C,D). In contrast, the incorporation of peptides lowered RMSD values of the complex, indicating a stabilizing effect on the T1R1-Glu and T1R3-Glu complexes. Greater structural stability is associated with enhanced umami intensity, which may explain the umami-enhancing properties of these peptides. The RMSD fluctuation values of all complexes were less than 1 nm in a reasonable fluctuation range, suggesting that the structure of the receptor–ligand complex was balanced in the simulation [57]. Further, compared with reported RMSD values for other peptide complexes [13,15], the receptor–peptide systems in this study exhibited lower RMSD values (0.4–0.6 nm), indicating higher structural stability and stronger binding affinity between the umami peptides (GAAGAAD, HQADGKS, and MPPTEPECEK) and receptors.

The RMSF curve quantifies the flexibility of individual amino acid residues in the receptor throughout the molecular dynamics simulation. It also reflects the flexibility and motion intensity of amino acids on the receptor [58]. The RMSF analysis indicated that fluctuations were within 0.8 nm for the T1R1-peptide complexes, 1.4 nm for the T1R3–peptide complexes, and 1 nm for the T1R1/T1R3-Glu-peptide complexes. Specifically, the RMSF values of most of the amino acids were within 0.4 nm (Figure 8E–H). All of those indicated that the receptor protein maintained relative stability throughout the simulation. Most of the higher RMSF values for all complexes were observed at residues 200–600 and 800–850, similar to the findings of Lao et al. [15]. These regions exhibit significant structural fluctuations, suggesting that these were the active regions of T1R1/T1R3. Notably, the T1R3-HQADGKS complex and T1R3-MPPTEPECEK complex had maximum fluctuation with 1.3 nm (residues 477–489) and 1.2 nm (residues 800–850), respectively, indicating heightened flexibility in these specific regions (Figure 8F). Furthermore, compared with the RMSF values of T1R1-Glu and T1R3-Glu (Figure 8G,H), the addition of peptides obviously lowered the RMSF values of most amino acids, including regions that initially exhibited high flexibility. This decrease suggests that umami peptides improve amino acid engagement with T1R1/T1R3, thereby strengthening overall receptor–ligand stability [13].

In addition, Rg often indicates protein structure compactness [43]. Similar to RMSD, the Rg values stabilized after 40 ns of simulation (Figure 8I–L), indicating that the peptides formed tight complexes with all receptors. The Rg values of the complex of the GAAGAAD and MPPTEPECEK fluctuated within 0.15 nm, while HQADGKS fluctuated within 0.5 nm (Figure 8I–J). This suggests that the T1R1/T1R3-GAAGAAD and T1R1/T1R3-MPPTEPECEK complexes exhibited greater structural stability. Interestingly, the Rg fluctuation ranges of the T1R1-Glu–peptide and T1R3-Glu–peptide complexes were all 0.1 nm. These results indicated that the receptor-Glu-peptide complex was more stable, thereby exhibiting greater umami intensity. This may be the reason for the stronger umami-enhancing ability. More minor Rg fluctuations generally indicate that the peptide has less influence on protein structural changes [15]. In our study, the Rg values of the T1R1/T1R3-Glu–peptide complexes ranged from 2.9 to 3.2 nm (Figure 8K,L). Compared with previous studies that found that the Rg values of T1R1/T1R3-MSG–peptide ranged from 5.4 to 6.2 [13], the Rg values of T1R1/T1R3-Glu-peptide in this study were significantly lower, indicating that the peptide had less influence on the receptor protein. Furthermore, the T1R1-Glu complexes and T1R3-Glu complexes fluctuated between 3.0 and 3.1 nm and 2.9–3.2 nm, respectively, and stabilized at 3.2–3.1 nm (Figure 8K,L). In contrast, the addition of the peptide not only reduced the Rg values but also significantly reduced the fluctuation range of the complex. These results indicate that the addition of this peptide was able to maintain structural compactness and minimize conformational alterations in T1R1/T1R3, which may also account for its umami-enhancing effect. Taken together, the peptide had the lowest impact on receptor stability, thereby confirming the receptor-peptide’s structural integrity and supporting the results of the molecular docking analysis.

## 4. Conclusions

In conclusion, four umami peptides—GAAGAAD, HQADGKS, GDDDEVEAAM, and MPPTEPECEK—were successfully identified and screened using UPLC-MS/MS and online tools. These peptides exhibited both umami taste and umami-enhancing effects, with GAAGAAD showing the strongest activity. Furthermore, they significantly enhanced saltiness while suppressing bitterness in the MSG solution. The peptides mainly interacted with the VFT region of T1R1/T1R3 via hydrogen bonding, forming stable complexes. The peptide bound more stably to the T1R1/T1R3-Glu complex, likely due to a significant increase in the number of interactions and hydrogen bonds. Notably, the results found that the residues of T1R1 (Cys50, Cys106, and Ser148) and T1R3 (Ser66, Ser67, Arg180, Asp215, Val277, and His278) might be new potential active sites. The MDS results further revealed the stability and tight binding of peptides to their receptors, thereby confirming the reliability and accuracy of the molecular docking results. Overall, these findings not only advance the molecular understanding of umami perception and umami enhancement but also support the potential use of umami peptides in seasoning formulations and sodium-reduced foods.

## Data Availability

The data that support the findings of this study are available from the corresponding author upon reasonable request due to privacy concerns.

## Supplementary Materials

The following supporting information can be downloaded at: https://www.mdpi.com/article/10.3390/foods15040680/s1, Table S1. Identification and virtual screening of umami peptides from soy sauce; Table S2. Molecular docking energies and binding sites of T1R1/T1R3 docking with umami peptides; Table S3. Molecular docking energies and binding sites of T1R1/T1R3-Glu docking with umami peptides; Figure S1 Schematic diagram of the virtual screening for potential umami peptides from soy sauce; Figure S2 Taste characteristics odor of soy sauce peptides (A) and their combined taste characteristics with sodium glutamate (MSG) (B) by electronic tongue; Figure S3 Interactions between Glu and T1R1 (A) and T1R3 (B) and the number of interactions (C) by molecular docking.
