# Supplementary material for: Identification and Characterization of Novel Umami and Umami-Enhancing Peptides from Soy Sauce Using an In Silico Approach and Electronic Tongue
Source: Foods. 2026 Feb 12;15(4):680. doi: 10.3390/foods15040680 (PMC12939475; doi:10.3390/foods15040680)
Supplement: Supplementary file 1 [file foods-15-00680-s001.zip › foods-4114657-supplementary.pdf]

Table S1. Identification and virtual screening of umami peptides from soy sauce.

Peptide sequences	Mass	Length	m/z	D and E frequency (%)	iUmami-SCM	UMPred-FRL	Umami-YYDS	Umami-MRNN	ToxinPred	AllerTOP
HTPFFK	776.41	6	388.71	0.00	534.00	0.02	0.97	Umami	Non-toxin	Non-Allergen
AKAARPR	811.49	7	406.25	0.00	571.67	0.37	1.00	Umami	Non-toxin	Non-Allergen
DYRIRKH	986.54	7	494.29	14.29	546.50	0.85	0.55	Umami	Non-toxin	Non-Allergen
GAAGAAD	531.23	7	532.25	14.29	631.83	0.54	1.00	Umami	Non-toxin	Non-Allergen
HQADGKS	741.34	7	742.38	14.29	639.50	0.61	1.00	Umami	Non-toxin	Non-Allergen
KLLLHRR	935.63	7	468.32	0.00	551.00	0.30	0.00	Non-Umami	Non-toxin	Allergen
KPPRPTK	822.51	7	823.53	0.00	557.00	0.11	1.00	Umami	Non-toxin	Non-Allergen
KTVSLPR	842.51	7	421.76	0.00	540.83	0.84	1.00	Umami	Non-toxin	Non-Allergen
SEWDRRH	984.45	7	493.26	28.57	600.33	0.47	1.00	Umami	Non-toxin	Non-Allergen
SGLSGGG	533.24	7	534.26	0.00	541.67	0.07	1.00	Umami	Non-toxin	Non-Allergen
YGGFLDK	841.41	7	421.21	14.29	507.50	0.09	0.97	Umami	Non-toxin	Allergen
HETHEGVQ	935.41	8	468.72	25.00	585.71	0.03	1.00	Umami	Non-toxin	Non-Allergen
MFDLHADK	976.46	8	488.73	25.00	608.57	0.05	1.00	Umami	Non-toxin	Allergen
VATVSLPR	842.51	8	421.76	0.00	545.29	0.69	1.00	Umami	Non-toxin	Non-Allergen
ELAAATNQF	963.47	9	482.75	11.11	582.88	0.94	1.00	Umami	Non-toxin	Non-Allergen
NQSSGADER	963.41	9	482.21	22.22	647.88	0.61	1.00	Umami	Non-toxin	Allergen
APAIVLGFEC	1018.52	10	510.27	10.00	545.00	0.59	0.08	Umami	Non-toxin	Non-Allergen
FEAEVYVLSK	1184.62	10	592.82	20.00	620.78	0.40	1.00	Umami	Non-toxin	Non-Allergen
GDDDEVEAAM	1092.39	10	547.21	50.00	674.56	0.78	1.00	Umami	Non-toxin	Non-Allergen
GKKVVVVGFK	1060.69	10	530.85	20.00	580.44	0.83	0.00	Umami	Non-toxin	Allergen
IVLYRLPYFT	1325.74	10	663.90	0.00	577.00	0.04	1.00	Non-Umami	Non-toxin	Non-Allergen
MPPTEPECEK	1217.52	10	609.26	30.00	593.89	0.90	1.00	Umami	Non-toxin	Non-Allergen

Table S2. Molecular docking energies and binding sites of T1R1/T1R3 docking with umami peptides.

	Peptide sequences	Binding energy (kcal/mol)	The sites of the hydrogen bond	The sites of hydrophobic interaction	The sites of electrostatic interaction
T1R1					
	GAAGAAD	-7.60	Ser48, Asp108, Asp147, Asn150, Asp218, Ser276, Arg277, Gln278		Asp218
	HQADGKS	-8.70	Ser48, Gly49, Asp147, Asn150, Ala170, Ser217, Asp218, Ser276, Arg277, Gln278, Ala302		Arg277, His71, Asp192, Glu301
	GDDDEVEAAM	-8.80	Ser107, Asp108, Asp147, Ser148, Thr149, Asn150, Arg151, Ser217, Phe247, Ser276, Arg277, Ala302, Ser385,	Pro45, His71, Arg277, Arg307,	Arg277, Tyr220
	MPPTEPECEK	-7.90	Leu51, Asp108, Asp147, Ser148, Arg151, Ser248, Ser384, Ser385	Cys50, Leu51, Val251, Leu279	Arg151
T1R3					
	GAAGAAD	-8.20	Glu45, Ser104, His145, Gly168, Tyr218, Val277, His278, Glu301		Arg180, Asp190, His278, Glu301
	HQADGKS	-9.00	Glu45, Ala46, Asn68, His145, Gly168, Arg180, Leu245, Pro246, Arg247, Asp249, Ser276, His278, Ala302, Gln389	His278	Glu148, His145, Asp216
	GDDDEVEAAM	-9.10	Ser67, Ser104, Pro106, His145, Ser147, Glu148, Arg180, Leu245, Ala248, Ser276, Val277, His278, Gln389	His145, Tyr218, Ala302	His145, Arg180, Asp249, His278
	MPPTEPECEK	-8.10	Arg180, Gln181, Tyr182, Gln221, Ser276 , His278	Pro42, His145, Leu308, Leu245,	Arg220, His278

Table S3. Molecular docking energies and binding sites of T1R1/T1R3−Glu docking with umami peptides.

	Peptide sequences	Binding Energy (kcal/mol)	The sites of the hydrogen bond	The sites of hydrophobic interaction	The sites of electrostatic interaction
T1R1−Glu					
	GAAGAAD	-8.0	Gly49, Asn150, Ser276, Arg277, Asp147, Val105, Asp108, Ser48, Ser148, Asp218, Cys106	Cys50, Leu279	Arg151
	HQADGKS	-8.8	Glu1, Gly49, Cys50, Leu51, Ser148, Asn150, Arg277, Asp147, Ser385, Pro45, Phe247, Gly49, Cys106, Ser276	Ala302, Leu279	Arg151
	GDDDEVEAAM	-8.9	Asp147, Pro45, Cys106, Cys50, Ser148, Asn150, Phe247, Ser276, Ser384, Ser385, Ser48, Ser248	Leu305, Met383, Arg277	Asp108, Glu1, Lys155
	MPPTEPECEK	-8.0	Glu1, Cys50, Gln52, HIS71, Ser148, Ser217, Ser276, Gln278, Leu61, Ser48, Cys66, Ser67	Arg277, Ala302, Met383, His71	Arg54, Glu1, Asp147, Asp63
T1R3−Glu					
	GAAGAAD	-8.6	Arg180, Ser66, Ser67, Ser104, Val277, Glu45, His278, Phe65, Ala46	Val277, Leu308, Val277, Leu245, Pro246, His278	His278
	HQADGKS	-9.2	Glu1, Arg180, Asn68, Ser276, Val277, His278, Ser104, Glu45, Asp215, Leu245, Asp216, Ala302, Ser66, Glu105, Pro106, Ser146, Arg247	His278, Val277, Leu245, Leu245, Pro246	
	GDDDEVEAAM	-9.2	Asp307, Arg180, Ala46, Ser66, Ser67, Asn68, His145, Asp249, Val277, His278, Leu308, Glu148, Asp216, Pro246, Ser276, Asp215	Pro246, Leu245	Asp307, Glu1, Arg64, His145
	MPPTEPECEK	-8.3	His145, Arg180, Ser67, Asp249, Val277, Asp307, His278, Asn68, Glu45, Ser104, Asp215, Pro106	Pro42, Ala383, Trp72, His145	His145, Glu1, Arg180, Asp307, Glu148

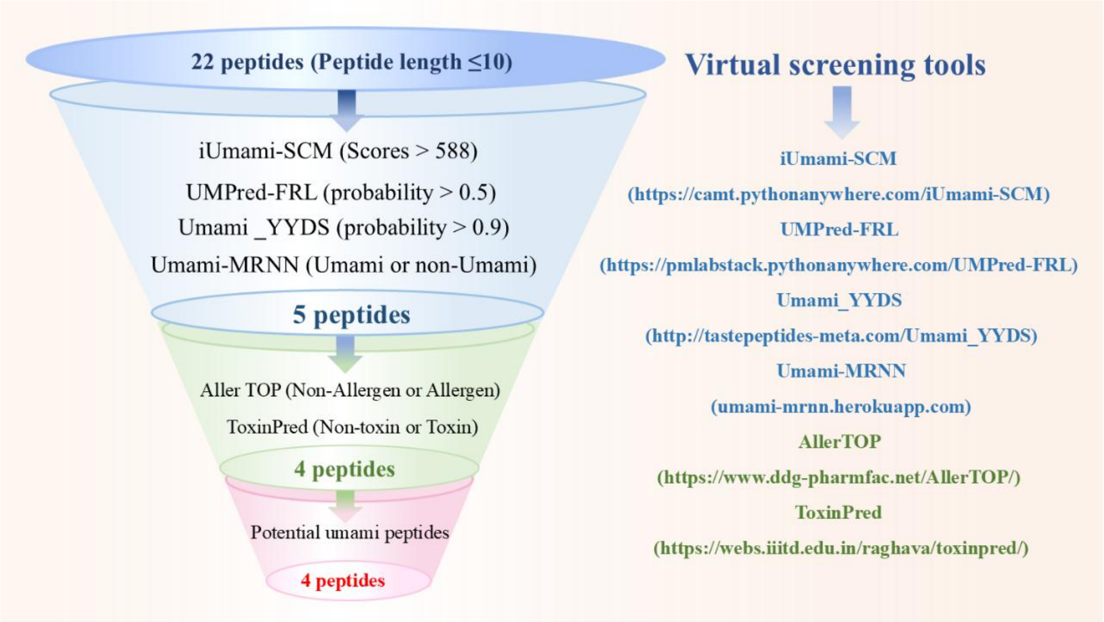

Figure S1 Schematic diagram of the virtual screening for potential umami peptides from soy sauce

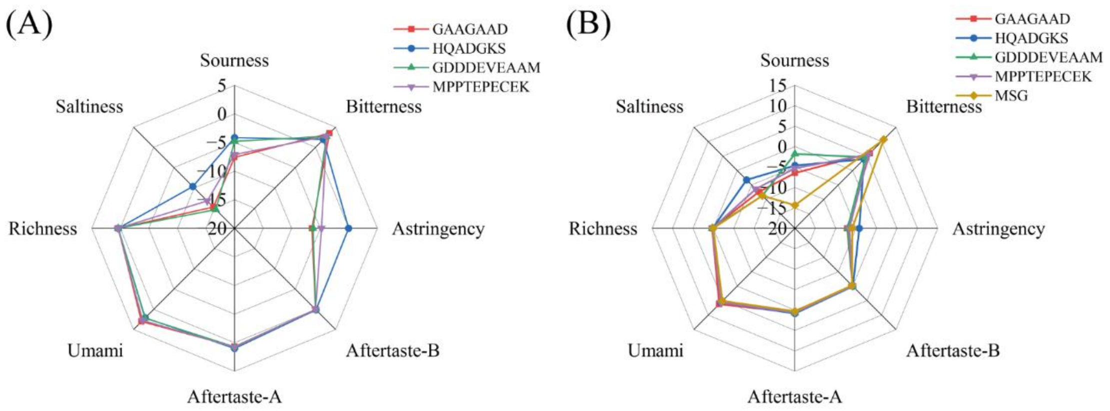

Figure S2 Taste characteristics odor of soy sauce peptides (A) and their combined taste characteristics with sodium glutamate (MSG) (B) by electronic tongue.

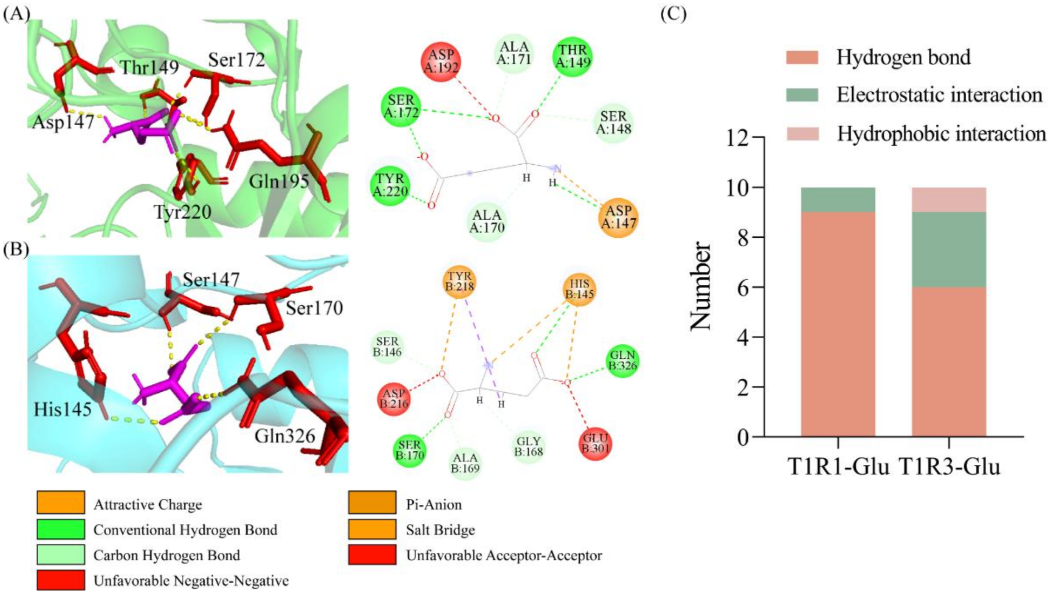

Figure S3 Interactions between Glu and T1R1 (A) and T1R3 (B) and the number of interactions (C) by molecular docking.
